# HPLC-UV method development and validation for anticancer drug sorafenib and the co-prescribed drug dexamethasone: application to pharmacokinetic evaluation of sorafenib nanoformulations

**DOI:** 10.3389/fphar.2025.1442762

**Published:** 2025-04-09

**Authors:** Abdul Mateen, Abad Khan, Ismail Khan, Saifullah Khan Khalil, Lateef Ahmad, Muhammad Junaid, Saqib Jehan, Muhammad Sohail Anwar, Muhammad Faheem, Abdul Salam

**Affiliations:** ^1^ Department of Pharmacy, University of Swabi, Swabi, Pakistan; ^2^ HBS Institute of Healthcare and Allied Health Sciences, Islamabad, Pakistan; ^3^ Department of Pharmacy, Sarhad University of Science and Information Technology, Peshawar, Pakistan; ^4^ Institute of Pathology and Diagnostics Medicine, Khyber Medical University, Peshawar, Pakistan

**Keywords:** HPLC, nanoformulation, sorafenib, dexamethasone, spiked plasma

## Abstract

Sorafenib is used to treat advanced renal cell carcinoma. A high-performance liquid chromatography (HPLC) method was developed for the simultaneous determination of sorafenib with a commonly co-prescribed drug, dexamethasone, using meloxicam as an internal standard. The separation was achieved with acetonitrile and water with 0.05% trifluoroacetic acid (TFA), 65:35 v/v, eluted at 1.0 mL/min at a wavelength of 265 nm. The chromatographic separation was carried out on an ACE Generic C18 (5 μm, 4.6 mm × 150 mm, UK) column by injecting a sample volume of 20 µL into the HPLC system. The analytes were extracted with acetonitrile using the protein precipitation method. The chromatographic parameters were optimized, and the method was validated as per the International Council for Harmonisation of Technical Requirements for Pharmaceuticals for Human Use (ICH) guidelines. The internal standard concentration was kept constant (1.0 μg/mL) in all samples. The method was linear for both sorafenib and dexamethasone in the concentration ranges of 25–1,000 ng/mL and 50–2,000 ng/mL, respectively, with a correlation coefficient (r^2^) of 0.999 for both the analytes. The target compounds were well resolved within 8 min. The limits of detection (LODs) are 9 ng/mL and 14 ng/mL, while the limits of quantification (LOQs) are 26 ng/mL and 47 ng/mL for sorafenib and dexamethasone, respectively. The method was found to be accurate and precise with a percentage relative standard deviation (RSD) of less than ±15. The method was successfully applied to evaluate the pharmacokinetics of a sorafenib nanoformulation and a conventional formulation. The AUC_0-t_ was significantly increased for the sorafenib nanoformulation (129.8 ± 1.54 µg-hrml^−1^) compared to the conventional formulation (15.0 ± 0.014 µg-hrml^−1^), while clearance was reduced for the sorafenib nanoformulation (31.551 ± 0.007 mlh^−1^kg^−1^) compared with the conventional formulation (426.856 ± 0.098 mlh^−1^kg^−1^).

## 1 Introduction

Cancer is a common cause of death in both developed and developing countries (Jemal et al., 2011). Hepatocellular carcinoma (HCC) is the fifth most prevalent cancer worldwide, and it represents the third most common cancer-related death worldwide, accounting for more than 0.5 million deaths per year (Michielsen et al., 2005). Development of HCC is a gradual process starting from decreased expression of tumor suppressor genes and increased expression of oncogenes leading to sequential increase activation of Raf/MEK/extracellular signal-regulated kinase (ERK) pathways (Whittaker et al., 2010).

Sorafenib was first approved by the US Food and Drug Administration (FDA) for the treatment of advanced renal cell carcinoma in 2005 and for HCC in 2007 (Keating and Santoro, 2009). Sorafenib is a novel small-molecule inhibitor of a variety of tyrosine kinase receptors, such as VEGFR-2, VEGRF-3, and PDGF-ß receptors, and it inhibits serine/threonine kinases as well. The action of VEGFR, PDGFR, and Rakinases, when not correctly regulated, may cause carcinogenesis (Wilhelm et al., 2004). Certainly, previous studies have suggested that sorafenib is able to inhibit the growth of tumor cells by blocking main signaling pathways that control cancer cell proliferation and angiogenesis (Wilhelm et al., 2008). Various commercially available formulations of sorafenib include Nexavar^®^ Tablets, oil suspension of sorafenib tosylate, Ora-Sweet^®^ solutions, Ora-Plus^®^ suspension, and extemporaneously compounded capsules of sorafenib tosylate (Navid et al., 2013). Currently, the recommended dose of sorafenib (Nexvar^®^) tablets is 400 mg twice daily, even though repeated dose reductions or discontinuation of therapy take place because of the intolerable adverse effects, such as hypertension, gastrointestinal irritation (even frequent episodes of bleeding), nausea, diarrhea, and fatigue (Mancuso et al., 2011).

The development of sorafenib-loaded-nanoparticle formulations can overcome the reported drawbacks. Nanoparticles can control drug release rates and target the delivery of sorafenib to the tumor tissues (Wang et al., 2011). Nanoparticles consist of an inner hydrophobic core acting as a reservoir for hydrophobic drugs, surrounded by a hydrophilic shell. Due to their unique structure, nanoparticles are considered an ideal vehicle for targeting hydrophobic drugs to the tumor. Nanoparticles can be broadly classified into several categories: magnetic nanoparticles, gold nanoparticles, quantum dots, micelles, dendrimers, polymeric nanoparticles, liposomes, and carbon nanotubes. Most FDA-approved nanoparticle formulations are liposomal nanomedicines (e.g., Doxil^®^, Myocet^®^, and DaunoXome^®^) (Zhang et al., 2014).

Dexamethasone is an anti-inflammatory and immunosuppressive agent used to manage several diseases like asthma, rheumatoid arthritis, various inflammatory and autoimmune disorders, allergic reactions, and organ transplant rejection (Lazar and Culver, 2010). Most chemotherapeutic treatments are mitigated by the addition of dexamethasone and glucocorticoids (GCs) (Kemeny et al., 2003; Rutz and Herr, 2004). GCs are co-prescribed with cytotoxic drugs to treat tumors, inflammation, pain, electrolyte imbalance, or toxic reactions associated with cancer therapy (Rutz and Herr, 2004; Rutz, 2002).

**FIGURE 1:**
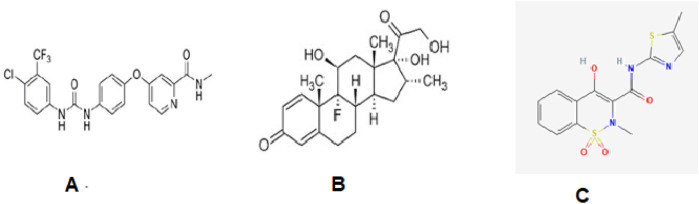
Structures of sorafenib **(A)**, dexamethasone **(B)**, and meloxicam **(C)**.

The purpose of the current study is to design a simple, inexpensive, and reproducible high-performance liquid chromatography (HPLC) method for the concurrent detection of sorafenib, dexamethasone, and meloxicam (Figure 1) in human plasma and apply the proposed method for the pharmacokinetic analysis of a sorafenib nanoformulation.

## 2 Materials and methods

### 2.1 Chemical substances, reagents, and internal standard

Sorafenib (purity ≥99.9%) was received from Qi Lu Laboratories, China. The dexamethasone and meloxicam were a gift from Nenza Pharmaceutical, Pakistan. Acetonitrile (ACN), methanol (MeOH), dichloromethane (DCM), trifluoroacetic acid (TFA), and polyvinyl alcohol were purchased from Sigma-Aldrich, Germany, while poly(lactic-co-glycolic acid (PLGA) (75:25), with a molecular weight of 76,000–115,000, was obtained from Evonik Germany. The Dialysis tubing used was obtained from Sigma-Aldrich. Chitosan with low- and high-molecular weight (reagent grade) was used as a coating polymer. Distilled water was used to prepare stock solutions and their respective dilutions.

### 2.2 Equipment

An HPLC system (Sykam Germany), linked with a pump, a UV–visible detector, and a vacuum degasser, was used in the study. The chromatographic separation was achieved using the ACE Generic C18 (5 μm, 4.6 × 150 mm, UK) column.

Chromatographic separation was carried out with ACN and TFA (0.05%), 65:35 v/v, eluted at 1.0 mL/min at a wavelength of 265 nm. The sample (20 µL) was analyzed by the system. Meloxicam was used as the internal standard (IS) to compensate for the possible loss of analytes during analysis.

### 2.3 Standard solution

The stock solutions (100 μg/mL) of the drugs and IS were made in ACN and further diluted with 25–1,000 ng/mL of the mobile phase for sorafenib, 50–2,000 ng/mL for dexamethasone, and 1,000 ng/mL for the IS. The solutions were stored at −20°C.

### 2.4 Sample preparation

**FIGURE 2:**
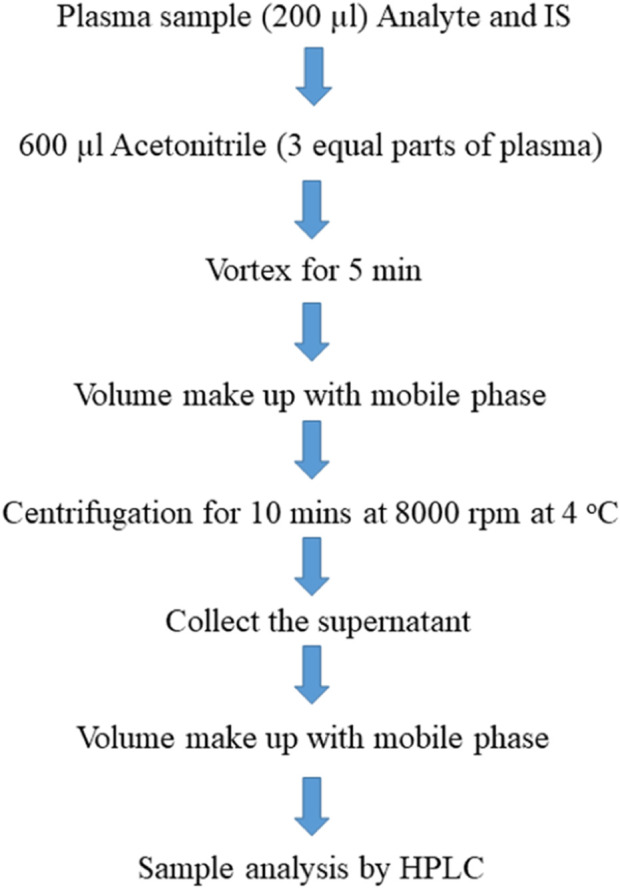
Scheme of sample preparation.

The plasma was separated from the blood by centrifugation at 8,000 rpm for 10 min at −4°C. The plasma (200 µL) was spiked with sorafenib, dexamethasone, and IS, the protein was precipitated with acetonitrile, and the extraction of the analytes was carried out with ACN, MeOH, and DCM. The following plan was applied to obtain the sample (Figure 2), and a sample (20 µL) was applied for analysis.

### 2.5 Preparation and characterization of nanoparticles

Sorafenib nanoparticles were synthesized through the solvent evaporation method (Yang et al., 2009) using PLGA and chitosan as polymers and Tween 80 as a stabilizer. The polymer concentration was kept constant, while the drug and surfactant (Tween 80) proportions were changed. The optimized nanoformulation (TWC60) was prepared in the composition of sorafenib 1 mg, PLGA 5 mg, and Tween 80 1% (13 mL). The optimized formulation was fabricated with chitosan 0.2% (2 mL) for surface modification. The physicochemical properties of the nanoformulations were characterized. The analysis of sorafenib in the nanoparticle formulation and pharmacokinetic profiling in animal plasma was carried out using the developed method.

### 2.6 Experimental parameters

The HPLC parameters were optimized, including the stationary phase (ACE Generic C18 column and Supelco Discovery HS C18 column) and solvents, including methanol, acetonitrile, and TFA, in different compositions as a mobile phase at different flow rates and at different wavelengths (260–275 nm) for simultaneous quantification of the target compounds.

### 2.7 Method validation

The accuracy was authenticated on the basis of the percentage recovery method at three different concentrations. Plasma (200 µL) was spiked with 0.025 μg/mL, 0.400 μg/mL, and 0.800 μg/mL of sorafenib and 0.05 µg/mL, 0.80 µg/mL, and 1.60 μg/mL of dexamethasone, respectively, while IS was kept constant. The readings were taken in triplicate, and % recovery was calculated by applying Equation 1.

$$\%\;Recovery=\frac{A}{B}\;X\;100.$$ (1)

A is the concentration in plasma, and B is the concentration in the mobile phase.

The specificity/selectivity was achieved through the complete separation of the analytes in the subject samples. The precision determined on the basis of the repeatability of injections was evaluated by injecting the plasma samples spiked with drugs and IS 10 times into the HPLC system. The %RSD is the measure of precision. Similar analysis repeatability was carried out on five plasma samples spiked with drugs and IS. The intraday and interday precision were performed for 1 day and 1 week, respectively, using Equation 2.

$$C=\frac{X}{Y}\;X\;\frac{A}{B}\;X\;FS\;X\;FD,$$ (2)

where X and Y are the ratios of the analyte in plasma and a 1:1 mixture, while A and B are the ratios of IS in a 1:1 mixture and plasma, respectively; FS is the concentration in the 1:1 mixture; FD is the dilution factor.

Linearity was confirmed through least squares regression, while sensitivity was measured on the basis of the S/N ratio of HPLC software. The S/N ratios are 3 and 10 for LOD and LOQ, respectively. Similar robustness was tested through planned changes in parameters while stability studies were performed at different storage conditions (‒20°C, 4°C, and 25°C) for a month using Equation 3.

$$\%\;Stability=\frac{St}{So}\;X\;100,$$ (3)

where St indicates stability at time t, and S0 indicates stability at time 0.

### 2.8 Pharmacokinetic parameter evaluation

The pharmacokinetic parameters were evaluated in New Zealand white rabbits weighing between 1,500 gm and 2,000 gm. The Ethical Committee approved the study design by letter No. Pharm/EC/035. The drug was administered in a dose of 10 mg/kg body weight, and the blood samples were taken at intervals of 0.25 h, 0.5 h, 1 h, 2 h, 4 h, 6 h, 8 h, 12 h, 24 h, 36 h, 48 h, 72 h, 96 h, 120 h, 144 h, 168 h, 192 h, 216 h, and 240 h. The samples were centrifuged and analyzed by HPLC. Different pharmacokinetic parameters like elimination half-life (t_1/2_), peak plasma concentration (C_max_), area under the plasma concentration-versus-time curve (AUC0-∞), clearance (CL), area under the curve (AUC), volume of distribution (V_d_), and mean residence time (MRT) were measured with the help of PK-Summit software (Summit Research Services, Pharmacokinetics and Metabolism software, PK Solutions 2.0) applying non-compartmental analysis.

### 2.9 Release kinetics

The release kinetics of the developed formulation were determined using the dialysis bag method (Mateen et al., 2024).

### 2.10 Application of the method

The developed method is part of a project involving the evolution of sorafenib nanoparticles in dosage form and plasma. The method will be useful for the concurrent quantification of sorafenib, dexamethasone, and meloxicam in dosage forms and plasma. The nanoformulation developed for sorafenib and conventional formulations were administered to rabbits weighing not less than 1 kg. The blood samples were collected at different time intervals and were analyzed through the developed HPLC method. The pharmacokinetic parameters Cmax, AUC, MRT, elimination half-life, volume of distribution, and clearance were determined using PK-Summit.

## 3 Results

**FIGURE 3:**
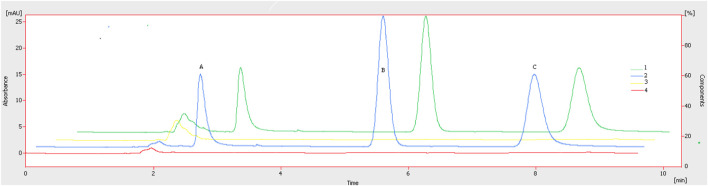
Representative chromatograms. A = Dexamethasone (1.6 µg), B = meloxicam (1.0 µg), and C = sorafenib (0.8 µg). Peaks: 1, spiked plasma; 2, standard solution; 3, blank plasma; 4, blank, respectively. Mobile phase = ACN: TFA (0.05%), flow rate = 1.0 mL/min, wavelength = 265 nm.

**FIGURE 4:**
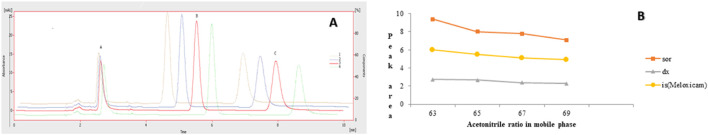
Effect of acetonitrile (%). 1 = 69%, 2 = 67%, 3 = 65%, 4 = 63%. Peaks A, B, and C are dexamethasone, meloxicam, and sorafenib, respectively. Mobile phase = ACN: TFA (0.05%), flow rate = 1.0 mL/min, wavelength = 265 nm. **(A)** Overlay chromatogram and **(B)** Line graph.

**FIGURE 5:**
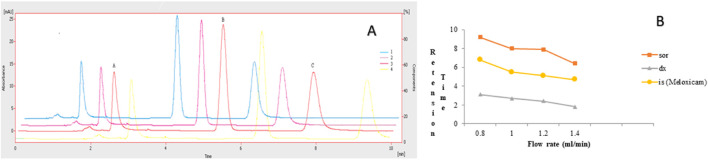
Effect of flow rate. 1 = 1.4:2 = 1.2:3 = 1.0:4 = 0.8 mL/min: Peaks A, B, and C are dexamethasone, meloxicam, and sorafenib, respectively. Mobile phase = ACN: TFA (0.05%), wavelength = 265 nm. **(A)** Overlay chromatogram and **(B)** line graph.

**FIGURE 6:**
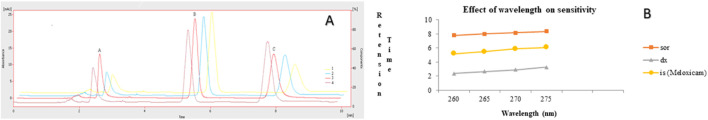
Effect of wavelength. 1 = 275 nm, 2 = 270 nm, 3 = 265 nm, 4 = 260 nm. Peaks A, B, and C are dexamethasone, meloxicam, and sorafenib, respectively. Mobile phase = ACN: TFA (0.05%), flow rate = 1.0 mL/min. **(A)** Overlay chromatogram and **(B)** line graph.

The chromatographic method was developed to quantify sorafenib, dexamethasone, and meloxicam (IS) and validated as per standard guidelines (Khan et al., 2010). The representative overlay chromatograms are shown in Figure 3. Among the different organic solvents evaluated for the extraction of target compounds, acetonitrile showed better results. A Thermo HS C18 column was selected from the tested columns as it gave better results, as shown in Figure 4. The mobile phase comprising acetonitrile and TFA (0.05%), 65:35 v/v was optimized, as shown in Figure 4. The flow rate of 1.0 mL/min and wavelength of 265 nm were adjusted for optimum retention, and better responses were recorded (Figures 5, 6). The compatibility of meloxicam with target compounds was optimum.

**FIGURE 7:**
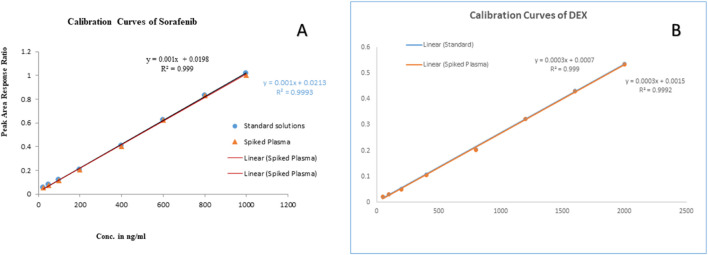
Linearity curves of standard solutions and plasma: **(A)** sorafenib, and **(B)** dexamethasone.

**FIGURE 8:**
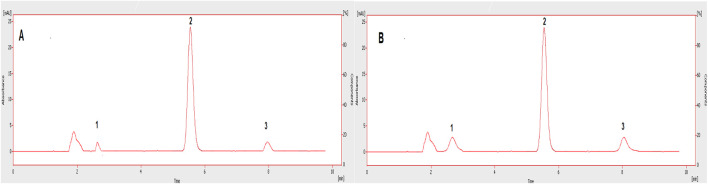
Chromatograms showing LOD **(A)** and LOQ **(B)** of the tested drugs in plasma. 1: Dexamethasone, 2: IS, 3: Sorafenib.

The linearity of the method was evaluated in the range, keeping in mind the minimum and maximum plasma concentrations of the studied drugs. The linearity of sorafenib (25–1,000 ng/mL) and dexamethasone (50–2,000 ng/mL) in standard solution and spiked plasma was confirmed as presented in Table 1; Figure 7. The accuracy of the method is presented in Table 2, while the precision and sensitivity data are shown in Table 3, 4. The sensitivities (LOD and LOQ) for both drugs are shown in Figure 8. It was determined that small, deliberate changes have no impact on the method. Stability studies showed that samples were stable for 72 h, as shown in Table 5.

**TABLE 1:** Linearity and accuracy.

Parameters	Sorafenib	Dexamethasone	
Linearity			
Concentration range (ng/mL)	25–1,000	50–2,000	
S. Solutions			
R. Equation	y = 0.001x + 0.0213	y = 0.0003x + 0.0015	
C. Coefficient, *r*	0.9993	0.9992	
S. Plasma			
R. Equation	y = 0.001x + 0.0198	y = 0.0003x + 0.0007	
C. Coefficient, *r*	0.999	0.999	
% Recovery			
25 ng/mL	98.10 ± 0.70; 0.71	50 ng/mL	98.13 ± 0.70; 0.72
400 ng/mL	98.73 ± 0.34; 0.35	800 ng/mL	98.56 ± 0.40; 0.41
800 ng/mL	99.72 ± 0.19; 0.19	1,600 ng/mL	99.04 ± 0.14; 0.14
Amount Recovered			
25 ng/mL	0.024 ± 0.0002; 0.84	50 ng/mL	0.049 ± 0.0002; 0.40
400 ng/mL	0.398 ± 0.0008; 0.22	800 ng/mL	0.789 ± 0.0013; 0.16
800 ng/mL	0.798 ± 0.0006; 0.08	1,600 ng/mL	1.598 ± 0.0011; 0.06

**TABLE 2:** Validation parameters.

Injection repeatability (ng/mL)	Sorafenib	Repeatability injection repeatability (ng/mL)	Dexamethasone
Retention time (min)	8.01 ± 0.01; 0.17	Retention time (min)	2.69 ± 0.002; 0.09
Peak area, 800 ng/mL	244.83 ± 1.60; 0.65	Peak area, 1,600 ng/mL	127.16 ± 1.47; 1.15
Analysis repeatability		Analysis repeatability	
Amount recovered (800)	0.79 ± 0.0005; 0.66	Amount recovered (1,600)	1.59 ± 0.0008; 0.05
Sensitivity			
Limit of detection (ng/mL)	9	Limit of detection (ng/mL)	14
Limit of quantification (ng/mL)	26	Limit of quantification (ng/mL)	47

**TABLE 3:** Precision studies.

Parameters	Analytes	Added Amount (ng/mL)	Recovered (ng/mL)	± SD	Precision (% RSD)	Accuracy (%)
Intraday	Sorafenib	25	24	0.09	0.41	96.0
		400	398	0.59	0.15	99.5
		800	798	0.63	0.08	99.8
	Dexamethasone	50	49	0.28	0.58	98.0
		800	789	1.97	0.25	98.6
		1,600	1,597	0.95	0.06	99.8
Interday	Sorafenib	25	24	0.32	1.35	96.0
		400	395	3.16	0.80	98.8
		800	791	4.42	0.56	98.9
	Dexamethasone	50	49	0.49	1.02	98.0
		800	787	5.43	0.69	98.4
		1,600	1,596	3.67	0.23	99.8

**TABLE 4:** Stability in terms of % recovery of sorafenib-spiked plasma samples stored at different temperatures.

Plasma Storage condition	Storage Time (hours)	% Recovery (Mean ± SD)		
		250 ng/mL	500 ng/mL	1,000 ng/mL
25°C	0	98.15 ± 0.44; 0.45	98.25 ± 0.55; 0.56	98.55 ± 0.19; 0.19
	6	97.45 ± 0.34; 0.35	97.39 ± 0.12; 0.12	98.05 ± 0.15; 0.15
	24	95.51 ± 0.53; 0.55	96.05 ± 0.41; 0.43	97.55 ± 0.26; 0.27
	72	85.90 ± 0.50; 0.58	84.95 ± 0.34; 0.40	88.25 ± 0.74; 0.84
4°C	6	98.13 ± 0.19; 0.19	98.29 ± 0.65; 0.66	98.91 ± 0.91; 0.92
	24	98.11 ± 0.38; 0.77	98.30 ± 0.67; 0.68	98.05 ± 0.68; 0.69
	72	97.36 ± 0.85; 0.87	96.99 ± 0.32; 0.33	96.93 ± 0.48; 0.50
Freeze and thaw 3x		96.41 ± 0.17; 0.18	97.05 ± 0.22; 0.23	98.00 ± 0.87; 0.89

**TABLE 5:** *In vitro* release of the optimized nanoformulations.

Formulation Code	Zero order	First order	Higuchi	Hixson–Crowell	Korsmeyer–Peppas	
	(R^2^)	(R^2^)	(R^2^)	(R^2^)	(R^2^)	(n*)
F4 (60TWC)	0.8912	0.6156	0.9866	0.9262	0.9784	0.50

The compounds were separated with better sensitivity (Figure 3).

### 3.1 *In vitro* evaluation of sorafenib nanoformulations

**FIGURE 9:**
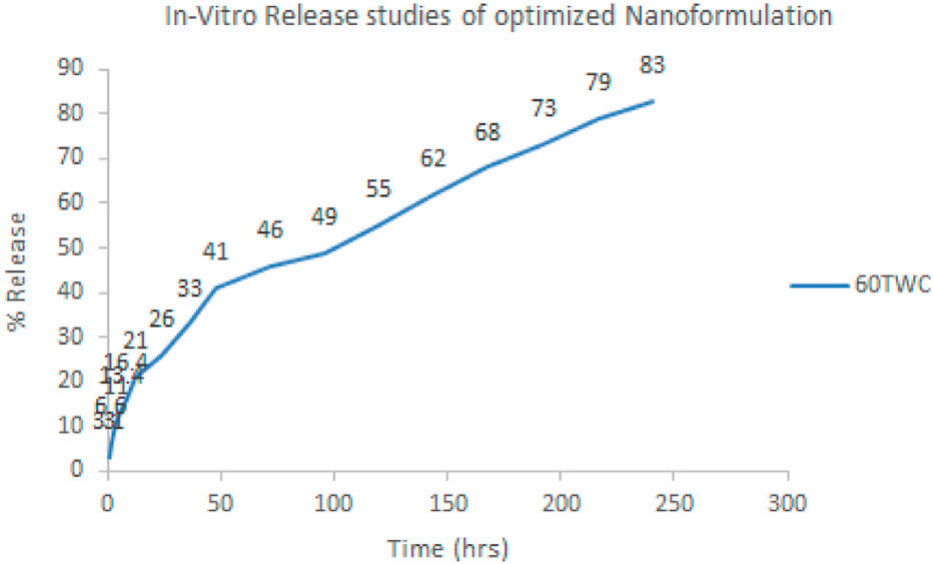
*In vitro* release profile of optimized nanoformulation.

The *in vitro* evaluation of sorafenib nanoformulations was carried out using the dialysis bag to determine the release mechanism and duration, as shown in Figure 9. A 1-mL sample of the nanoformulation was dialyzed against 100 mL of PBS (pH 7.2) at 37°C and 60 rpm. The sample (1 mL) was withdrawn at fixed intervals (0.25 h, 0.5 h, 1 h, 2 h, 4 h, 6 h, 8 h, 12 h, 24 h, 36 h, 48 h, 72 h, 96 h, 120 h, 144 h, 168 h, 192 h, 216 h, and 240 h) and was analyzed in triplicate to determine the drug release. The volume was replaced with the dissolution media.

Various models were applied to the proposed formulations to identify the release kinetics of the drug and release mechanism from the dosage forms, as given in Table 5 and Figure 9. Diffusion followed by erosion has taken place in the proposed formulation as indicated by the “R” and “n” values.

### 3.2 Pharmacokinetic evaluation

**FIGURE 10:**
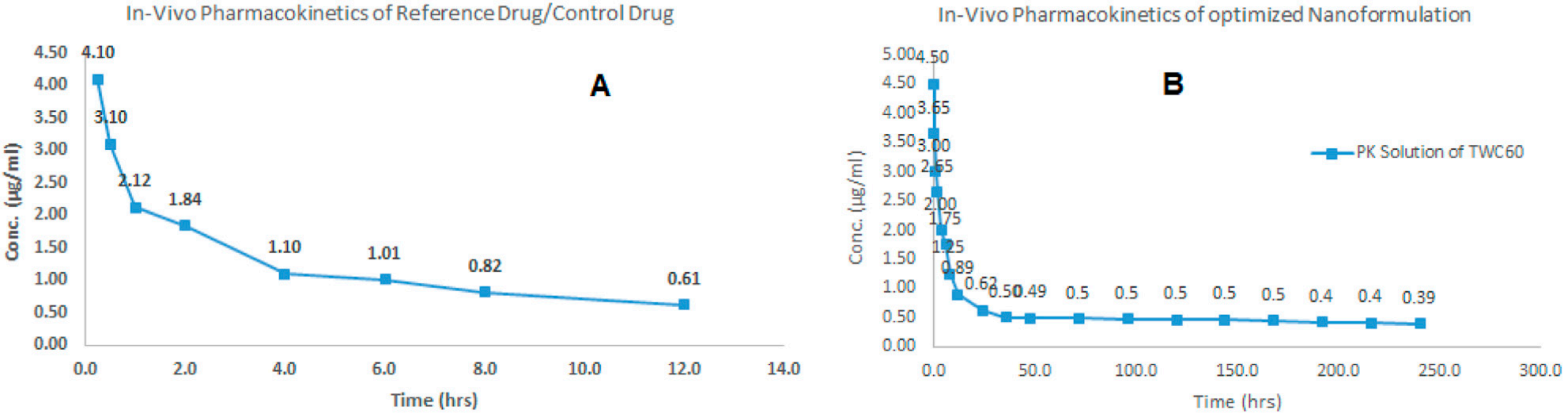
*In vivo* profile of sorafenib: **(A)** conventional formulation and **(B)** nanoformulation.

The pharmacokinetic profiling of sorafenib nanoformulation was performed in rabbits. The pharmacokinetic parameters were measured (Table 6; Figure 10) by applying PK-Summit^®^. The C_max_ values of sorafenib in conventional and nanoparticle formulations are 4.1 μg/mL and 4.5 μg/mL, respectively. The elimination half-life increased from 9.54 h to 332.51 h, AUC increased from 15 µg-h/L to 129.8 µg-h/L, AUMC increased from 282.3 µg-h×h/L to 148,955.3 µg-h×h/L, mean residence time increased from 12 h to 465.4 h, V_d_ increased from 5881.1 L to 15138.5 L, and clearance decreased from 426.856 L/h to 31.551 L/h in both formulations, respectively.

**TABLE 6:** Pharmacokinetic parameters of sorafenib.

Parameters	Dose (IV)	C_max_	AUC_0−t_	AUMC∞	MRT	t ½	Vd	CL
Unit	mg kg^−1^	µg ml^−1^	µg-hrml^−1^	µg-hr×hrml^−1^	hr	hr	ml	mlh^−1^kg^−1^
References/control	10.00 ± 00	4.1 ± 0.011	15.0 ± 0.014	282.3 ± 1.12	12.0 ± 0.05	9.54 ± 0.14	5881 ± 1.56	426.8 ± 0.098
60TWC	10.00 ± 01	4.5 ± 0.012	129.8 ± 1.54	148,955 ± 735	465 ± 1.37	332 ± 6.67	15138 ± 0.56	31.5 ± 0.007
*p*-value	—	—	0.001***	0.001***	0.001***	0.001***	0.001***	0.001***

**FIGURE 11:**
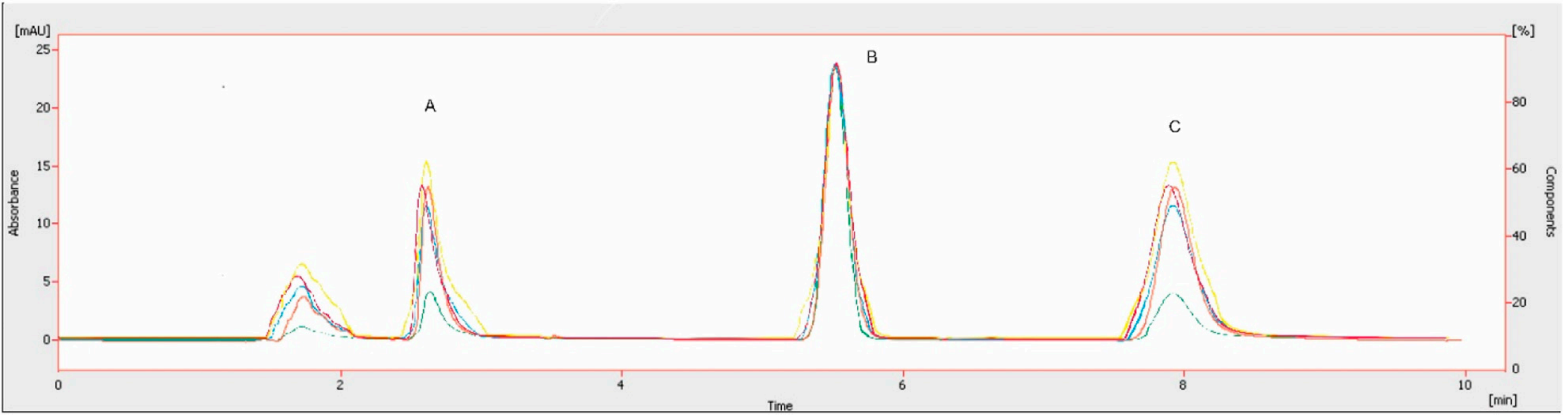
Overlay chromatograms of plasma sample with A: dexamethasone, B: IS, C: sorafenib.

The overlay chromatogram of the tested compounds is given in Figure 11. The comparison of the proposed method with the reported methods is given in Table 7.

**TABLE 7:** Comparison of the proposed method with the reported method.

Parameters	Proposed Method	Reported Method (Ismail et al., 2016)	Reported Method (Blanchet et al., 2009)	Reported Method (Sharkawi et al., 2023)
Mobile phase	ACN and TFA (0.05%) 65:35 v/v	Acetonitrile and TFA (0.025%) in the ratio of (65:35 V/V)	Acetonitrile/20 mM ammonium acetate (60:40)	Methanol: 40 mM ammonium acetate solution (pH = 5.5)
Limit of detection	9 ng/mL	15 ng/mL		
Limit of quantification	26 ng/mL	10 ng/mL	0.5 mg/L	0.03 ug/mL
Linearity	25–1,000 ng/mL	5–500 ng/mL	0.5–20 mg/L	0.03–30 μg/mL
Run time	08 min	12 min	14 min	08 min
Recovery	>98%	95%	87%	
Simultaneous Drug	Dexamethasone	Paclitaxel		Gemcitabine
IS	Meloxicam	Piroxicam	Erlotinib	Sildenafil

### 3.3 Application

**FIGURE 12:**
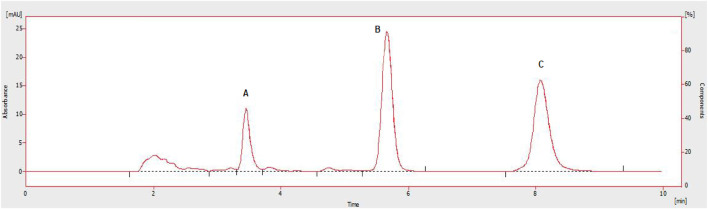
Chromatograms showing C_max_ rabbit plasma concentrations of administered drugs A: dexamethasone, B: IS, C: sorafenib.

This method is part of an *in vitro* and *in vivo* evaluation of sorafenib nanoformulations. The *in vitro* release studies were carried out to measure the release pattern and mechanism of sorafenib from a polymeric nanoformulation, while the pharmacokinetic studies of sorafenib nanoparticles were conducted in animals using the proposed HPLC method (Figure 12). The method can be utilized to quantify sorafenib, dexamethasone, and meloxicam in dosage forms and in other biological fluids.

## 4 Discussion

The simultaneous analysis of sorafenib and dexamethasone is reported for the first time using meloxicam as an IS applying the proposed method. The compounds were eluted in 10 min with good resolution and higher sensitivity. The optimization of experimental parameters was made on the basis of good instrumental response. The acetonitrile and 0.05%TFA in water (65:35 v/v) were applied at a wavelength of 265 nm and eluted at 1.0 mL/min in isocratic mode.

The linearity was measured for sorafenib and dexamethasone. The LOD values for sorafenib and dexamethasone were 9 ng/mL and 14 ng/mL, while the LOQ values for sorafenib and dexamethasone were 26 ng/mL and 47 ng/mL, respectively. According to the ICH guidelines, the accuracy and precision (% RSD) values were within the range for the developed method. The samples stored at different temperatures for different time intervals demonstrated good stability. The release kinetics and pharmacokinetics parameters of sorafenib nanoformulations were evaluated using the proposed methods. The sorafenib nanoformulation release pattern was improved as compared to the conventional formulation. According to the R^2^ value, it was observed that the drug release from the 60TWC formulation best fits the Higuchi model, and release from polymer was described by diffusion, followed by erosion, and finally by n value, showing Fickian diffusion. A further *in vivo* bioavailability study was done that showed significantly increased bioavailability.

Compared with the reported methods (Khan et al., 2016; Blanchet et al., 2009; Sharkawi et al., 2023), the new method is simple, rapid, sensitive, and inexpensive. The LOQ of the method allows us to apply the method for the evaluation of the target compounds without using complex and expensive techniques like capillary electrophoresis or LC-MS-MS.

The limitation of the study was that only the pharmacokinetic parameters of sorafenib were determined, while the method was developed for the simultaneous determination of both sorafenib and dexamethasone. The release kinetics of the reference drug were followed for 10 h only. The linearity curve was constructed in the desired therapeutic levels rather than the LOQ.

## 5 Conclusion

The developed method was validated, and the experimental parameters were authenticated. The method has been applied for the simultaneous quantification of sorafenib and dexamethasone in standard solutions and plasma samples. The method was implemented in the analysis of sorafenib nanoformulation in rabbits. The presented method is fast, simple, and inexpensive for the concurrent analysis of both drugs in a single run.

## Data Availability

The original contributions presented in the study are included in the article/supplementary material; further inquiries can be directed to the corresponding author.
